# MSC-CSMC: A multi-objective semi-supervised clustering algorithm based on constraints selection and multi-source constraints for gene expression data

**DOI:** 10.3389/fgene.2023.1135260

**Published:** 2023-02-27

**Authors:** Zeyuan Wang, Hong Gu, Minghui Zhao, Dan Li, Jia Wang

**Affiliations:** ^1^ Faculty of Electronic Information and Electrical Engineering, Dalian University of Technology, Dalian, Liaoning, China; ^2^ Department of Breast Surgery, Second Hospital of Dalian Medical University, Dalian, Liaoning, China

**Keywords:** semi-supervised clustering, constraint selection, multi-source constraints, gene expression data, multi-objective optimization

## Abstract

Many clustering techniques have been proposed to group genes based on gene expression data. Among these methods, semi-supervised clustering techniques aim to improve clustering performance by incorporating supervisory information in the form of pairwise constraints. However, noisy constraints inevitably exist in the constraint set obtained on the practical unlabeled dataset, which degenerates the performance of semi-supervised clustering. Moreover, multiple information sources are not integrated into multi-source constraints to improve clustering quality. To this end, the research proposes a new multi-objective semi-supervised clustering algorithm based on constraints selection and multi-source constraints (MSC-CSMC) for unlabeled gene expression data. The proposed method first uses the gene expression data and the gene ontology (GO) that describes gene annotation information to form multi-source constraints. Then, the multi-source constraints are applied to the clustering by improving the constraint violation penalty weight in the semi-supervised clustering objective function. Furthermore, the constraints selection and cluster prototypes are put into the multi-objective evolutionary framework by adopting a mixed chromosome encoding strategy, which can select pairwise constraints suitable for clustering tasks through synergistic optimization to reduce the negative influence of noisy constraints. The proposed MSC-CSMC algorithm is testified using five benchmark gene expression datasets, and the results show that the proposed algorithm achieves superior performance.

## 1 Introduction

The rapid development of microarray technology has generated a large amount of gene expression data and mining the inherent patterns in the massive gene expression data is a major challenge in the current bioinformatics field (Bandyopadhyay et al., 2007; Pirooznia et al., 2008). As an important unsupervised data mining method, clustering has become a powerful tool for gene expression data analysis. One of the main tasks of gene expression data clustering is to identify co-expressed genomes, which is a useful tool for further research on gene function (Bandyopadhyay et al., 2007; Chen et al., 2019). Compared with the unsupervised clustering methods, the semi-supervised clustering methods use prior information to guide the clustering process through data labels or pairwise constraints, which can effectively improve the performance of clustering (Wagstaff et al., 2001; Bilenko et al., 2004; Yin et al., 2010).

For semi-supervised clustering algorithms, the pairwise constraints are usually used to describe if two data belong to the same cluster. Specifically, the must-link constraint (ML) means that two data must be divided into the same cluster, and the cannot-link constraint (CL) means that two data must be divided into different clusters. The quality of the selected pairwise constraints is of vital importance, which significantly affects the performance of semi-supervised clustering algorithms (Grira et al., 2008; Vu et al., 2012; Masud et al., 2019; Abin and Vu, 2020). The pairwise constraints can be generated by directly using part of the known data labels (Lai et al., 2021) or by using an active learning method (Masud et al., 2019). In practical, most gene expression data are unlabeled, for which it is impossible to obtain pairwise constraints based on labels. Vu et al. (2012) indicated that the generation of the pairwise constraints should mainly focus on the data samples on the cluster boundaries, which are more likely to be misclassified. To this end, Basu et al. (2004) developed a farthest-first traversal scheme-based active learning method to obtain pairwise constraints. However, this method has been reported to be sensitive to noise (Davidson and Qi, 2008). Grira et al. (2008) proposed an active learning method to generate pairwise constraints by determining cluster boundary data using membership obtained by fuzzy clustering. Vu et al. (2012) identified data in sparse regions based on *k*-nearest neighbor graphs and constructed pairwise constraints. However, it was claimed that some pairwise constraints might not be generated by this method (Abin and Vu, 2020). Liu et al. (2018) proposed an entropy-based query strategy to select the most uncertain pairwise constraints. Abin (2018) proposed a random walk approach on the adjacency graph of data for querying informative constraints. Masud et al. (2019) used local density estimation to identify the most informative objects as pairwise constraints. Abin and Vu (2020) proposed a density tracking method which takes into account the density relationship between data, and uses the information about boundaries and skeleton of clusters to generate the pairwise constraints.

Although the above methods can automatically mine and learn the pairwise constraints of unlabeled datasets through different approaches, there are inevitably noisy constraints, i.e., constraints inconsistent with the ground-truth clusters, in the obtained pairwise constraints (Yin et al., 2010; Lai et al., 2021). However, the existing semi-supervised clustering algorithms are mostly based on the assumption that pairwise constraints conform to real cluster information, and usually susceptible to noisy constraints. Therefore, it is necessary to implement constraints selection, where noisy constraints are filtered out, and only pairwise constraints that are beneficial for semi-supervised clustering are retained. In addition, most of the pairwise-constraints-based semi-supervised clustering algorithms were developed for single-source constraints, i.e., the pairwise constraints are obtained only from the data itself. In real-world applications, many data also possess related domain information. For example, Gene Ontology (GO) (Ashburner et al., 2000), which describes gene products in terms of their associated biological processes, cellular components and molecular functions, can further provide gene annotation information for gene expression data. In this paper, the multi-source constraints are the pairwise constraints formed by the data itself and domain information. Apparently, compared with the single-source pairwise constraints based solely on gene expression data, the multi-source constraints formed by the fusion of gene ontology can provide more comprehensive information about the structure of gene clusters and help to guide semi-supervised clustering to obtain more accurate clustering results.

Aiming at the unlabeled gene expression data and from the perspective of reducing the negative impact of noisy constraints and integrating multi-source constraints, a method called multi-objective semi-supervised clustering algorithm based on constraints selection and multi-source constraints (MSC-CSMC) is proposed in this research. At first, the proposed algorithm uses gene expression data and GO information to generate multi-source pairwise constraints. Then, under the multi-objective optimization framework of Non-dominated Sorting Genetic Algorithm-II (NSGA-II), the constraints selection and the cluster prototypes are collaboratively optimized to realize the selection of pairwise constraints suitable for clustering with respect to the multi-source constraints and to improve the accuracy of semi-supervised clustering of gene expression data by reducing the negative impact of noisy constraints.

## 2 Methods

**FIGURE 1:**
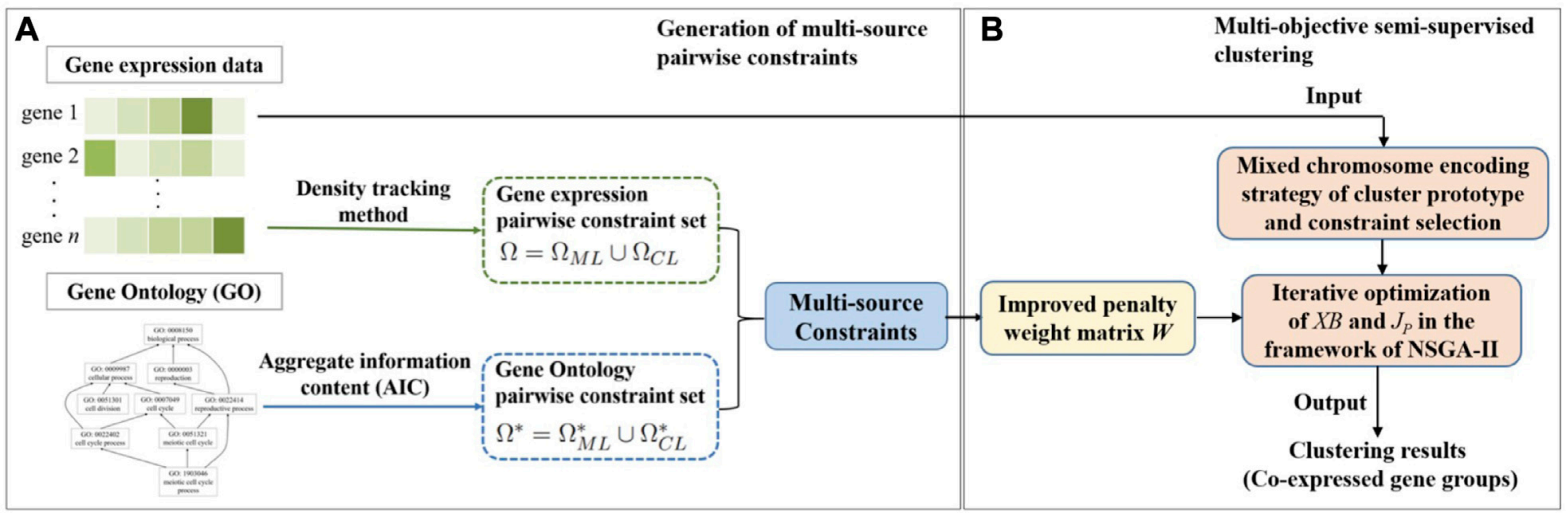
Workflow of MSC-CSMC. **(A)** Generation of multi-source pairwise constraints. **(B)** Multi-objective semi-supervised clustering.

In this section, the details of our proposed MSC-CSMC algorithm are described. Our proposed method consists of two parts. Firstly, multi-source pairwise constraints are generated by integrating gene expression and gene ontology (GO) information. Then, by using the improved penalty weights as well as mixed chromosome encoding strategy of cluster prototype and constraints selection, multi-objective semi-supervised clustering based on constraints selection and multi-source constraints is performed to identify co-expressed gene groups. The workflow of MSC-CSMC is shown in Figure 1.

### 2.1 Generation of multi-source pairwise constraints

Gene expression data and gene ontology (GO) describe gene-related information from the abundance of mRNA of genes and gene annotation. Compared with the method only using gene expression data, the combination of these two aspects of information can help to further improve the clustering accuracy of gene expression data (Giri and Saha, 2020; Li et al., 2022). In this paper, we use gene expression data and gene ontology information to generate multi-source pairwise constraints for semi-supervised clustering.

In view of the superior performance of the density tracking method (Abin and Vu, 2020), we use this method to generate the initial gene expression constraint set. The method consists of three steps: density estimation, density following, and constraints generation. Let $X=\left\{x_{1},\;x_{2},\ldots x_{n}\right\},\;x_{i}\in R^{d}$ denote a *d*-dimensional gene expression dataset with *n* genes. Gene ***x***_*i*_’s density is obtained by

$$Density\left(x_{i}\right)=\frac{1}{\underset{x_{j}\in N_{b}\left(x_{i}\right)}{\max}{\left\|x_{i}-x_{j}\right\|}_{2}},$$ (1)

where *N*_*b*_(***x***_*i*_) is the set of *b* nearest genes of gene ***x***_*i*_; ${\left\|\cdot \right\|}_{2}$ is the Euclidean distance. Based on the density in Formula 1, the density tracking method constructs density chains according to the density relationship between data. Specifically, starting from each gene ***x***_*i*_, the closest gene ***x***_*j*_ ∈ *N*_*b*_(***x***_*i*_) whose density is greater than that of ***x***_*i*_ is selected, and the relation between them is recorded as density chain ***x***_*i*_ → ***x***_*j*_. Then start from gene ***x***_*j*_ and continue the above density tracking until there exists no gene whose density is greater than that of the gene at the end of the chain. Consequently, the density chain *Chains* (***x***_*i*_) can be denoted as ***x***_*i*_ → ***x***_*j*_ → ⋯ → ***x***_*e*_. After constructing all the density chains, the total times of gene ***x***_*i*_ appearing in all the chains is referred to as centrality and denoted by *Centrality* (***x***_*i*_). The sum of centrality with respect to all genes in a density chain is used as the centrality of the density chain. All density chains with a common endpoint are considered connected density chains and the points belonging to them are considered to be in the same density group. Besides, the impurity of gene ***x***_*i*_ is defined as follows:

$$Impurity\left(x_{i}\right)=\left[1-\overset{\left|Groups\right|}{\underset{g=1}{\sum }}{\left(\frac{\underset{x_{j}\in S}{\sum }I\left(Group\left(x_{j}\right)=g\right)}{b+1}\right)}^{2}\right]\times \left[1-\frac{Density\left(x_{i}\right)}{Density\left(x_{e}\right)}\right]$$ (2)

with |*Groups*| being the total number of groups, *S* = {***x***_*i*_ ∪ *N*_*b*_(***x***_*i*_)}, *Group*(***x***_*j*_) being the group index of ***x***_*j*_, I being the indictor function.

**FIGURE 2:**
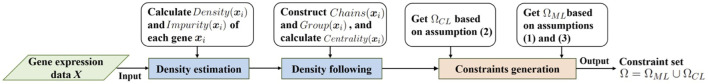
Workflow of density tracking method.

According to the density, impurity, density chain, and density group of the data, the density tracking method proposes three assumptions for mining informative pairwise constraints. Let Ω denote the pairwise constraint set, whose elements satisfy the following key assumptions: (1) providing feasible information about the boundary data of clusters; (2) providing feasible information about the boundary between various clusters; (3) providing feasible information about the skeleton of clusters. Among them, assumptions (1) and (3) are used to generate the must-link constraint set Ω_*ML*_, assumption (2) is used to generate the cannot-link constraint set Ω_*CL*_. With the subsets Ω_*ML*_ and Ω_*CL*_, the penalization can be constructed for the cost function of the clustering. The workflow of density tracking method is given in Figure 2. The initial gene expression constraint set Ω = Ω_*ML*_ ∪ Ω_*CL*_ is generated as follows.

1. For each gene ***x***_*i*_, calculate its *Density*(***x***_*i*_) and *Impurity*(***x***_*i*_). Construct density chain *Chains*(***x***_*i*_) and density group *Group*(***x***_*i*_), get the centrality of density chain. Initialize Ω_*ML*_ = ∅, Ω_*CL*_ = ∅;
2. Select gene ***x***_*i*_ in descending order of *Impurity* (***x***_*i*_), query the nearest neighbor gene ***x***_*j*_ that is not in its density group *Group* (***x***_*i*_), and add the pairwise constraint (***x***_*i*_, ***x***_*j*_) into the cannot-link constraint set, i.e., Ω_*CL*_ = Ω_*CL*_ ∪ {(***x***_*i*_, ***x***_*j*_)}.
3. Select gene ***x***_*i*_ in descending order of *Impurity*(***x***_*i*_), and find the next gene ***x***_*j*_ along its density chain *Chains*(***x***_*i*_). Let *ɛ* > 0 denote the density drop rate. If *Density*(***x***_*j*_) ≥*ɛ*× *Density*(***x***_*e*_), then add the pairwise constraint (***x***_*i*_, ***x***_*j*_) to the must-link constraint set, i.e., Ω_*ML*_ = Ω_*ML*_ ∪ {(***x***_*i*_, ***x***_*j*_)};
4. Select the density chain *Chains*(***x***_*i*_) in descending order of the centrality of the density chain, start from the starting gene ***x***_*i*_, select the gene ***x***_*j*_ with an interval, and add the pairwise constraint (***x***_*i*_, ***x***_*j*_) to the must-link constraint set, i.e., Ω_*ML*_ = Ω_*ML*_ ∪ {(***x***_*i*_, ***x***_*j*_)}.

For a set of genes to be analyzed, each gene can be annotated with several GO terms. Thus, the functional similarity between genes can be deduced based on the term similarity. In the proposed MSC-CSMC algorithm, we adopt the aggregate information content (AIC) (Song et al., 2014) to measure the semantic similarity of GO terms *t*_1_ and *t*_2_:

$$sim_{\mathrm{AIC}}\left(t_{1},t_{2}\right)=\frac{\sum_{t\in T_{t_{1}}\cap T_{t_{2}}}2\times SW\left(t\right)}{SV\left(t_{1}\right)+SV\left(t_{2}\right)}$$ (3)

with

$$SW\left(t\right)=\frac{1}{1+\exp\left(-1/IC\left(t\right)\right)},\;SV\left(t\right)=\underset{t^{\prime }\in T_{t}}{\sum }SW\left(t^{\prime }\right)$$

Here, *T*_*t*_ is the set of ancestors of term *t* in the GO graph, *p*(*t*) is the frequency of the term appearing in the GO database, *IC*(*t*) = − log  *p*(*t*) is the information content of term *t*. The higher the annotation frequency, the more general the information contained and the smaller the corresponding *IC* value. *SW*(*t*) normalizes the knowledge reflected by 1/*IC*(*t*), describing the semantic weight of term *t*. Consequently, the functional similarity of genes ***x***_*i*_ and ***x***_*j*_ can be obtained as follows:

$${sim}_{GO}\left(x_{i},x_{j}\right)=\frac{\underset{t_{2}\in ann\left(x_{j}\right)}{\sum }sim\left(x_{i},t_{2}\right)+\underset{t_{1}\in ann\left(x_{i}\right)}{\sum }sim\left(x_{j},t_{1}\right)}{\left|ann\left(x_{i}\right)\right|+|ann(x_{j})|}$$ (4)

where

$$sim\left(x_{i},t_{2}\right)=\underset{t_{1}\in ann\left(x_{i}\right)}{\max}\;{sim}_{AIC}\left(t_{1},t_{2}\right)$$

is the similarity of gene ***x***_*i*_ and term *t*_2_. *ann*(***x***_*i*_) and *ann*(***x***_*j*_) represent the sets of GO terms that annotate the two genes, respectively. The cardinalities of *ann*(***x***_*i*_) and *ann*(***x***_*j*_) are denoted by |*ann*(***x***_*i*_)| and |*ann*(***x***_*j*_)|, respectively.

The gene function similarity obtained through GO can also reflect the pairwise constraint relationship between genes to a certain extent. In the proposed MSC-CSMC algorithm, gene pairs with a similarity of more than 0.9 constitute the GO must-link constraint set $\Omega_{ML}^{*}$ , gene pairs with a similarity less than 0.1 constitute the GO cannot-link constraint set $\Omega_{CL}^{*}$ , and then generate the GO pairwise constraint set $\Omega^{*}=\Omega_{ML}^{*}\cup \Omega_{CL}^{*}$ . Finally, the gene expression pairwise constraint set Ω and the gene ontology pairwise constraint set Ω* together constitute multi-source constraints for gene clustering.

### 2.2 Semi-supervised clustering objective functions based on multi-source constraints

At present, multi-objective optimization has gradually become a mainstream method for solving gene expression data clustering problems, which can achieve better clustering results on gene expression data compared with single-objective optimization methods. In the unsupervised multi-objective clustering problem of gene expression data, the cluster validity indices *J*_*FCM*_ (Bezdek et al., 1981) and *XB* (Xie and Beni, 1991), which measure the intra-cluster compactness and inter-cluster separation respectively, are commonly used as objective functions to realize the evolution of decision variables based on two conflicting objectives (Bandyopadhyay et al., 2007; Maulik et al., 2009; Mukhopadhyay et al., 2013; Li et al., 2022). In this paper, the proposed MSC-CSMC algorithm uses *XB* and the function based on quadratic-regularized fuzzy c-means with constraint violation penalty, namely, *J*_*P*_ (Mei, 2019), as the objective functions. Furthermore, the constraint violation penalty weights in *J*_*P*_ are improved to achieve semi-supervised clustering of gene expression data based on the multi-source constraints in the NSGA-II framework. The objective functions of *XB* and *J*_*P*_ are as follows:

$$XB=\frac{\overset{k}{\underset{c=1}{\sum }}\overset{n}{\underset{i=1}{\sum }}u_{ic}^{2}{\left\|x_{i}-v_{c}\right\|}_{2}^{2}}{n\times \underset{f\neq c}{\min}{\left\|v_{f}-v_{c}\right\|}_{2}^{2}}$$ (5)

$$J_{P}=\overset{k}{\underset{c=1}{\sum }}\overset{n}{\underset{i=1}{\sum }}u_{ic}{\left\|x_{i}-v_{c}\right\|}_{2}^{2}+\frac{\eta }{2}\overset{k}{\underset{c=1}{\sum }}\overset{n}{\underset{i=1}{\sum }}u_{ic}^{2}-\frac{\beta }{2}\overset{n}{\underset{i=1}{\sum }}\overset{n}{\underset{j=1}{\sum }}w_{ij}u_{i}^{⊤}u_{j}$$ (6)

Here,

$$v_{c}=\frac{\sum_{i=1}^{n}u_{ic}x_{i}}{\sum_{i=1}^{n}u_{ic}}$$

is the *c*th cluster prototype. *k* is the number of clusters, parameters *η* and *β* control the level of fuzziness and the contribution of the penalty term during clustering, respectively. *u*_*ic*_ is the membership degree of the datum ***x***_*i*_ belonging to the *c*th cluster, obtained by

$$u_{ic}=\frac{1}{k}+\frac{1}{\eta }\left(u_{ic}^{FCM_{q}}+\beta u_{ic}^{P}\right)$$ (7)

$$u_{ic}^{FCM_{q}}=\frac{1}{k}\overset{k}{\underset{f=1}{\sum }}{\left\|x_{i}-v_{f}\right\|}_{2}^{2}-{\left\|x_{i}-v_{c}\right\|}_{2}^{2}$$ (8)

$$u_{ic}^{P}=\overset{n}{\underset{j=1}{\sum }}w_{ij}u_{jc}-\frac{1}{k}\overset{k}{\underset{f=1}{\sum }}\overset{n}{\underset{j=1}{\sum }}w_{ij}u_{jf}$$ (9)

where *w*_*ij*_ ∈ ***W*** is the penalty weight for violating pairwise constraint $\left(x_{i},x_{j}\right)$ . In order to simultaneously consider both the gene expression constraint set Ω = Ω_*ML*_ ∪ Ω_*CL*_ and gene ontology constraint set $\Omega^{*}=\Omega_{ML}^{*}\cup \Omega_{CL}^{*}$ , that is, the multi-source constraints proposed in this paper, we improve the constraint violation penalty weights through the following analysis: (1) if pairwise constraint $\left(x_{i},x_{j}\right)$ exists in both Ω_*ML*_ and $\Omega_{ML}^{*}$ , or in both Ω_*CL*_ and $\Omega_{CL}^{*}$ , it means that the same category information of gene pair $\left(x_{i},x_{j}\right)$ can be obtained from gene expression and gene annotation, so the weight of violating this constraint should be increased; (2) if pairwise constraint $\left(x_{i},x_{j}\right)$ exists in Ω_*ML*_ but not in $\Omega_{ML}^{*}$ , or exists in Ω_*CL*_ but not in $\Omega_{CL}^{*}$ , it indicates that the category information of gene pair $\left(x_{i},x_{j}\right)$ is not clear enough, thus the penalty weight *w*_*ij*_ should be decreased; (3) if pairwise constraint $\left(x_{i},x_{j}\right)$ exists in both Ω_*ML*_ and $\Omega_{CL}^{*}$ , or in both Ω_*CL*_ and $\Omega_{ML}^{*}$ , it should be regarded as a contradictory constraint and removed from the constraint sets Ω and Ω*. Based on the above idea, the MSC-CSMC algorithm proposed in this paper improves the constraint violation penalty weight as follows:

$$w_{ij}=\left\{\begin{array}{cc} 1-\theta , & \left(x_{i},x_{j}\right)\in \Omega_{ML}\text{ and }\left(x_{i},x_{j}\right)\notin \Omega_{ML}^{*}\\ -1+\theta , & \left(x_{i},x_{j}\right)\in \Omega_{CL}\text{ and }\left(x_{i},x_{j}\right)\notin \Omega_{CL}^{*}\\ 1+\theta , & \left(x_{i},x_{j}\right)\in \Omega_{ML}\text{ and }\left(x_{i},x_{j}\right)\in \Omega_{ML}^{*}\\ -1-\theta , & \left(x_{i},x_{j}\right)\in \Omega_{CL}\text{ and }\left(x_{i},x_{j}\right)\in \Omega_{CL}^{*}\\ 0, & \text{ otherwise } \end{array}\right.$$ (10)

with *θ* > 0 being the GO action parameter. It can be seen that the improved penalty weights can effectively integrate the gene expression and Gene Ontology information, and provide reasonable violation penalty for pairwise constraints in semi-supervised clustering.

### 2.3 Mixed chromosome encoding strategy used in MSC-CSMC

**FIGURE 3:**
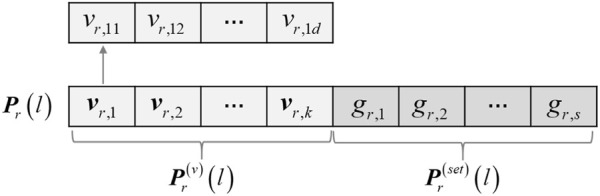
The mixed chromosome encoding strategy used in MSC-CSMC.

For the purpose of co-optimizing the constraints selection and clustering in the process of multi-objective evolution, a mixed encoding strategy combining the constraints selection and cluster prototype is adopted, as shown in Figure 3. Let ***P*** denote the genetic population, *N* be the population size, and *s* be the number of pairwise constraints to be selected. Considering the existence of noisy constraints in the initial pairwise constraint set and to improve the search efficiency of the algorithm, 2*s* constraints are randomly selected from the initial pairwise constraint set to generate the candidate constraint set Ω_*p*_, and a serial number is assigned for each pairwise constraint. For a gene expression dataset with *k* clusters $X=\left\{x_{1},\;x_{2},\ldots x_{n}\right\},\;x_{i}\in R^{d}$ , the *r*th individual in the *l*th generation $P_{r}\left(l\right)$ consists of two parts: the cluster prototype $P_{r}^{\left(v\right)}\left(l\right)$ and the constraints selection $P_{r}^{\left(set\right)}\left(l\right)$ . Among them, $P_{r}^{\left(v\right)}\left(l\right)=\left[v_{r,1},\;v_{r,2},\;\ldots ,\;v_{r,k}\right]$ encode *k* cluster prototypes $v_{r,c}=\left[v_{r,c1},\;v_{r,c2},\;\ldots ,\;v_{r,cd}\right]\left(1\leq c\leq k\right)$ with real numbers, $P_{r}^{\left(set\right)}\left(l\right)=\left[g_{r,1},\;g_{r,2},\;\ldots ,\;g_{r,s}\right]$ encode the serial numbers of *s* pairwise constraints $g_{r,j}\left(1\leq g_{r,j}\leq 2s,\;1\leq j\leq s\right)$ selected from Ω_*p*_ with integers.

In the proposed algorithm, the two parts of the chromosomes are initialized separately. For the cluster prototype part, in order to ensure initialization quality and population diversity, half of the individuals are encoded as the *k* cluster prototypes obtained by the density peak method (Rodriguez and Laio, 2014), and the other half are encoded from the randomly generated cluster prototypes. For the constraints selection part of each individual, the components are initialized with non-repeated random integers in $\left[1,2s\right]$ .

### 2.4 Genetic operations

In the genetic evolution process of the MSC-CSMC algorithm, the roulette wheel strategy is first used to implement the selection. Since the NSGA-II algorithm tends to select individuals with lower non-domination ranks, for the *r*th individual $P_{r}\left(l\right)$ of the *l*th generation, the selection probability (Zhou and Zhu, 2018) is calculated as follows:

$$p_{s}\left(P_{r}\left(l\right)\right)=\alpha {\left(1-\alpha \right)}^{f_{\mathrm{rank}}-1}$$ (11)

Here, $\alpha \in \left(0,1\right)$ is the selection parameter, *f*_*rank*_ is the non-domination rank of individual $P_{r}\left(l\right)$ .

For the parent individuals $P_{r_{1}}\left(l\right)$ and $P_{r_{2}}\left(l\right)$ , let the crossover probability be *p*_*c*_, different crossover operators are used for the cluster prototypes and constraints selection. Among them, $P_{r_{1}}^{\left(v\right)}\left(l\right)$ and $P_{r_{2}}^{\left(v\right)}\left(l\right)$ generate offspring through the normal distribution crossover operator (Zhang and Luo, 2009), and the offspring cluster prototypes are:

$$offsp_{1}^{\left(v\right)}=\frac{P_{r_{1}}^{\left(v\right)}\left(l\right)+P_{r_{2}}^{\left(v\right)}\left(l\right)}{2}+1.481\times \frac{P_{r_{1}}^{\left(v\right)}\left(l\right)-P_{r_{2}}^{\left(v\right)}\left(l\right)}{2}\times |N\left(0,1\right)|$$ (12)

$$offsp_{2}^{\left(v\right)}=\frac{P_{r_{1}}^{\left(v\right)}\left(l\right)+P_{r_{2}}^{\left(v\right)}\left(l\right)}{2}-1.481\times \frac{P_{r_{1}}^{\left(v\right)}\left(l\right)-P_{r_{2}}^{\left(v\right)}\left(l\right)}{2}\times |N\left(0,1\right)|$$ (13)

where $N\left(0,1\right)$ is a random variable of normal distribution. The constraints selection $P_{r_{1}}^{\left(set\right)}\left(l\right)$ and $P_{r_{2}}^{\left(set\right)}\left(l\right)$ adopts the single-point crossover operator, for a random integer *rand*_*c*_ in $\left[1,s\right]$ , the offspring constraints selections are:

$${offsp}_{1}^{\left(set\right)}=\left[g_{r_{1},1},\ldots ,g_{r_{1},{rand}_{c}},g_{r_{2},{rand}_{c}+1},\ldots ,g_{r_{2},s}\right]$$ (14)

$$off{sp}_{2}^{\left(set\right)}=\left[g_{r_{2},1},\ldots ,g_{r_{2},{rand}_{c}},g_{r_{1},{rand}_{c}+1},\ldots ,g_{r_{1},s}\right]$$ (15)

If repeated pairwise constraints appear after crossover, non-repeated pairwise constraints are randomly selected from the candidate constraint set Ω_*p*_ as a replacement. For individual ***P***_*r*_(*l*), different mutation operators are adopted for the two parts. The polynomial mutation operator (Rousseeuw, 1987) is applied for $P_{r}^{\left(v\right)}\left(l\right)$ , where site *v*_*r*,*ci*_ mutates with probability *p*_*m*_:

$$v_{r,ci}^{\prime }=v_{r,ci}+\delta \times \left(v_{u}-v_{l}\right),1\leq c\leq k,1\leq i\leq d$$ (16)

where, *v*_*u*_ and *v*_*l*_ are the upper and lower bounds of the cluster prototype, respectively. For normalized gene expression data, the bounds are set to 1 and 0. *δ* is determined as follows (Deb and Tiwari, 2008):

$$\delta =\left\{\begin{array}{c} {\left(2\times {rand}_{m}+\left(1-2\times {rand}_{m}\right){\left(1-v_{r,ci}\right)}^{\eta_{m}+1}\right)}^{\frac{1}{\eta_{m}+1}}-1,{rand}_{m}<0.5\\ 1-{\left[2\times \left(1-{rand}_{m}\right)+2\times \left({rand}_{m}-0.5\right)v_{r,ci}^{\eta_{m}+1}\right]}^{\frac{1}{\eta_{m}+1}},{rand}_{m}\geq 0.5 \end{array}\right.$$ (17)

Here, *η*_*m*_ is the distribution index, *rand*_*m*_ is a random number in $\left[0,1\right]$ . For $P_{r}^{\left(set\right)}\left(l\right)$ , random mutation is used, that is, first randomly select a position in $P_{r}^{\left(set\right)}\left(l\right)$ , and then replace its value with a random integer in $\left[1,2s\right]$ that is not repeated with others. In summary, the procedure of the MSC-CSMC algorithm is shown as follows:

Input: Gene expression dataset ***X***, number of neighbors *b*, density drop rate *ɛ*, population size *N*, maximal number of generations *L* *_max_*, number of clusters *k*, fuzzy parameter *η*, penalty parameter *β*, constraint number *s*, GO action parameter *θ*, selection parameter *α*, crossover probability *p*_*c*_, mutation probability *p*_*m*_, and distribution index *η*_*m*_.

Step 1: Generate gene expression pairwise constraint sets Ω based on density tracking method.
Step 2: Calculate the functional similarity of genes based on AIC, and generate the gene ontology pairwise constraint set Ω*. Then delete the contradictory constraints, and determine the penalty weight matrix ***W*** corresponding to the multi-source constraints based on Formula 10.
Step 3: Randomly select 2*s* pairwise constraints from the initial constraint set to construct the candidate constraint set Ω_*p*_, and initialize the population.
Step 4: When the genetic generation index is $l\left({l=1,2,\ldots ,L}_{\max}\right)$ , for each individual $P_{r}\left(l\right)\;\left(1\leq r\leq N\right)$ , decode to obtain the cluster prototypes and the selected pairwise constraints. Update the membership degree according to Formulas 7-9, and calculate the individual fitness values based on Formulas 5-6.
Step 5: According to the individual fitness values, calculate the non-domination rank and crowding distance of each individual.
Step 6: Apply selection, crossover, and mutation based on Formulas 11-17, and update the individual fitness values according to Formulas 5-6.
Step 7: Merge the parent and offspring populations, and select the next-generation according to the elite retention strategy.
Step 8: If $l=\left\lfloor 0.\;5\times L_{\max}\right\rfloor$ or $l=\left\lfloor 0.\;8\times L_{\max}\right\rfloor$ , update the penalty parameter *β* = 2 × *β* to increase the penalty for violating the currently selected constraints.
Step 9: Set *l* = *l* + 1, repeat Steps 4-8 until the maximal number of generations *L* *_max_* is reached.

Output: The Pareto optimal solutions.

## 3 Results

### 3.1 Datasets

In this study, five benchmark gene expression datasets, namely, Yeast Galactose Metabolism, Yeast Cell Cycle, Yeast Sporulation, Serum, and Arabidopsis are used for the experiment.

The Yeast Galactose Metabolism dataset (Ideker et al., 2001) is composed of 205 genes whose expression patterns reflect four functional categories. The gene expression profiles were measured with four replicate assays across 20 time points. The Yeast Cell Cycle dataset (Cho et al., 1998) contains the expression levels of 384 genes involved in yeast cell cycle regulation at 17 time points, and these data are related with five phases of cell cycle. The Yeast sporulation dataset (Chu et al., 1998) contains the expression levels of more than 6,000 genes measured during the sporulation process of budding yeast across seven time points. The genes that showed no significant changes in expression during the harvesting were excluded, and the resulting set consists of 474 genes. The Serum dataset (Iyer et al., 1999) contains the expression levels of 517 human genes. The dataset has 13 dimensions corresponding to 12 time points and 1 unsynchronized sample. The Arabidopsis dataset (Reymond et al., 2000) consists of 138 *Arabidopsis Thaliana* genes. Each gene has eight expression values that correspond to eight time points. The details of the datasets are shown in Table 1.

**TABLE 1:** Description of datasets.

Dataset	Number of genes	Number of features	Number of clusters
Yeast Galactose Metabolism	205	80	4
Yeast Cell Cycle	384	17	5
Yeast Sporulation	474	7	6
Serum	517	13	6
Arabidopsis	138	8	4

### 3.2 Model evaluation criteria and parameter assignment

In order to evaluate the effectiveness of the model, the silhouette index (Rousseeuw, 1987) is chosen as the evaluation criterion for the clustering results. For gene ***x***_*i*_, the silhouette width is calculated as follows:

$$S\left(i\right)=\frac{b\left(i\right)-a\left(i\right)}{\max\left\{a\left(i\right),b\left(i\right)\right\}},1\leq i\leq n$$ (18)

Here, *a*(*i*) is the average distance from gene ***x***_*i*_ to other genes in the same cluster, *b*(*i*) is the minimum average distance between gene ***x***_*i*_ and genes in the other clusters. The silhouette index *SI* of dataset ***X*** is the mean value of the silhouette widths of all genes, with $SI\in \left[-1,1\right]$ . A greater *SI* value represents the algorithm with better clustering quality. Besides, as suggested by (Saha and Bandyopadhyay, 2013), the final solution of MSC-CSMS is selected from Pareto optimal solutions by using the silhouette index.

**FIGURE 4:**
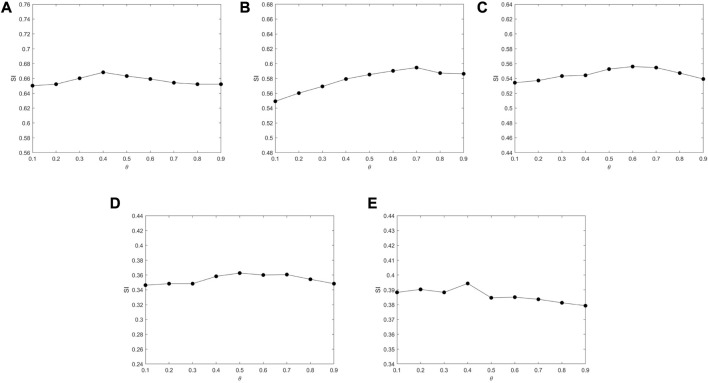
The impact of parameter *θ* on *SI* tested on different datasets. **(A)** Yeast Galactose Metabolism **(B)** Yeast Cell Cycle **(C)** Yeast Sporulation **(D)** Serum **(E)** Arabidopsis.

According to (Mei, 2019) and (Abin and Vu, 2020), the parameters of MSC-CSMC are assigned as follows: *ɛ* = 0.8, *b* = 10, *η* = 0.001, *β* = 0.1, *N* = 100, *L* *_max_* = 300, *α* = 0.3, *η*_*m*_ = 5, *p*_*c*_ = 0.8, *p*_*m*_ = 0.1. The number of pairwise constraints *s* is chosen as 0, 5, 10, 15, 20, and 25. In gene expression data analysis, the determination of the number of clusters *k* is an open problem. Generally, there are two approaches to determine the value of *k*; one is to directly set it as the true number of clusters (Yu et al., 2018; Zhao et al., 2021; Li et al., 2022; Liu et al., 2022; Wu and Ma, 2022); The other approach is applicable to the case where the true number of clusters is unknown, in which the variation range of *k* is determined firstly, and the *k* corresponding to the optimal value of an index (Silhouette index, Dunn index, Davies–Bouldin index, *etc.*) can be chosen as the optimal number of clusters (Gao et al., 2019; Acharya et al., 2020; López-Cortés et al., 2020; Zhang et al., 2022). In this paper, we adopt the first approach, and the number of clusters *k* is selected according to Table 1. In order to analyze the impact of the GO action parameter *θ*, we set *θ* from 0.1 to 0.9 at intervals of 0.1 under the condition that the number of the pairwise constraints is 15. The results are shown in Figure 4. It can be seen that the value of *SI* barely changes as *θ* increases, which means that the algorithm is not very sensitive to the value of *θ*. For Yeast Galactose Metabolism, Yeast Cell Cycle, Yeast Sporulation, Serum, and Arabidopsis, the *θ* values are respectively set to 0.4, 0.7, 0.6, 0.5, and 0.4, which lead to the optimal clustering performances.

### 3.3 Result analysis and model comparison

For the purpose of inspecting the performance of the proposed MSC-CSMC algorithm, several advanced semi-supervised clustering algorithms based on single-source constraints, including COP-Kmeans (Wagstaff et al., 2001), PCKMeans (Basu et al., 2004), MPCKMeans (Bilenko et al., 2004), PCCA (Grira et al., 2008), PCFCMq (Mei, 2019) and MSC-CS (Zhao and Li, 2022), are used for comparison. Among them, the MSC-CS algorithm is the single-source constrained version of MSC-CSMC, which does not consider the annotation information provided by GO. In the above algorithms, the pairwise constraints are randomly selected from the initial gene expression constraint set Ω. To avoid the influence of randomness, each method is run for ten times under the same number of pairwise constraints, and the mean value of the clustering results is taken as the final result. The *SI* values of all seven algorithms applied to five datasets are shown in Tables 2–6, the optimal solutions in each row are highlighted in bold.

**TABLE 2:** *SI* values on Yeast Galactose Metabolism with different number of constraints.

*s*	COP-Kmeans	PCKMeans	MPCKMeans	PCCA	PCFCMq	MSC-CS	MSC-CSMC
0	0. 384	0. 254	0. 305	0.525	0. 465	**0. 566**	**0. 566**
5	0. 423	0. 479	0. 258	0.348	0. 254	0. 583	**0. 628**
10	0. 460	0. 484	0. 471	0.144	0. 274	0. 592	**0. 631**
15	0. 458	0. 484	0. 463	0.198	0. 402	0. 645	**0. 668**
20	0. 459	0. 457	0. 370	0.383	0. 351	0. 645	**0. 668**
25	0. 445	0. 433	0. 413	0.351	0. 290	0. 645	**0. 668**

The bold values indicate the optimal solutions in each row.

**TABLE 3:** *SI* values on Yeast Cell Cycle with different number of constraints.

*s*	COP-Kmeans	PCKMeans	MPCKMeans	PCCA	PCFCMq	MSC-CS	MSC-CSMC
0	0. 256	0. 252	0. 281	0.350	0. 408	**0. 436**	**0. 436**
5	0. 264	0. 250	0. 251	0.115	0. 385	0. 456	**0. 497**
10	0. 273	0. 227	0. 203	0.208	0. 409	0. 519	**0. 542**
15	0. 258	0. 275	0. 202	0.133	0. 408	0. 528	**0. 594**
20	0. 282	0. 263	0. 322	0.229	0. 408	0. 530	**0. 606**
25	0. 264	0. 261	0. 318	0.267	0. 409	0. 584	**0. 607**

The bold values indicate the optimal solutions in each row.

**TABLE 4:** *SI* values on Yeast Sporulation with different number of constraints.

*s*	COP-Kmeans	PCKMeans	MPCKMeans	PCCA	PCFCMq	MSC-CS	MSC-CSMC
0	0. 329	0. 328	0. 345	0.400	0.364	**0. 491**	**0. 491**
5	0. 331	0. 354	0. 411	0.067	0.463	0. 520	**0. 528**
10	0. 324	0. 429	0. 404	0.164	0.420	0. 525	**0. 531**
15	0. 300	0. 404	0. 409	0.325	0.434	**0. 565**	0. 556
20	0. 324	0. 403	0. 405	0.235	0.416	0. 571	**0. 592**
25	0. 346	0. 396	0. 394	0.286	0.413	0. 592	**0. 594**

The bold values indicate the optimal solutions in each row.

**TABLE 5:** *SI* values on Serum with different number of constraints.

*s*	COP-Kmeans	PCKMeans	MPCKMeans	PCCA	PCFCMq	MSC-CS	MSC-CSMC
0	0. 212	0. 208	0. 186	0.290	0.270	**0. 312**	**0. 312**
5	0. 210	0. 205	0. 211	0.080	0.264	**0. 327**	0. 325
10	0. 200	0. 202	0. 197	0.146	0.271	**0. 341**	0. 340
15	0. 200	0. 181	0. 184	0.235	0.264	0. 354	**0. 362**
20	0. 198	0. 206	0. 217	0.144	0.262	0. 368	**0. 385**
25	0. 193	0. 202	0. 185	0.238	0.269	0. 379	**0. 403**

The bold values indicate the optimal solutions in each row.

**TABLE 6:** *SI* values on Arabidopsis with different number of constraints.

*s*	COP-Kmeans	PCKMeans	MPCKMeans	PCCA	PCFCMq	MSC-CS	MSC-CSMC
0	0. 220	0. 223	0. 197	0.314	0.353	**0. 358**	**0. 358**
5	0. 207	0. 216	0. 192	-0.151	0.353	0. 368	**0. 373**
10	0. 212	0. 210	0. 206	0.046	0.353	0. 373	**0. 387**
15	0. 200	0. 201	0. 185	0.106	0.354	0. 375	**0. 394**
20	0. 197	0. 189	0. 185	0.308	0.344	0. 381	**0. 396**
25	0. 187	0. 187	0. 181	0.335	0.352	0. 389	**0. 397**

The bold values indicate the optimal solutions in each row.

According to Tables 2–6, it can be seen that the proposed MSC-CSMS algorithm and its single-source constraint version MSC-CS can always achieve optimal and suboptimal clustering results on five gene expression datasets, demonstrating the effectiveness of the constraints selection. The mixed chromosome encoding strategy combining the constraint selection and cluster prototype can find the pairwise constraints suitable for clustering in the co-evolution process and improve clustering accuracy, and the highly accurate clustering results can further improve the constraint selection ability of the algorithm in turn. Conversely, the algorithms for comparison are based on the assumption that the pairwise constraints conform to the real cluster information and are easily affected by noisy constraints. This is consistent with the analysis of the negative effects of noisy constraints by (Yin et al., 2010) and (Lai et al., 2021). In addition, the MSC-CSMC algorithm is better than MSC-CS in most cases, indicating that using multi-source constraints can improve the performance of semi-supervised clustering. The gene ontology used to generate multi-source pairwise constraints in our MSC-CSMC algorithm can explain gene expression profiles from the perspective of gene function. By effectively integrating the gene expression and Gene Ontology information, the proposed penalty weights can provide reasonable violation penalty for pairwise constraints.

In the case of *s* = 0, that is, there is no pairwise constraint, both MSC-CSMC and MSC-CS degenerate into unsupervised multi-objective clustering methods, turning out the same result. Compared with PCFCMq, which uses *J*_*P*_ as the single objective function, the better performance of MSC-CSMC and MSC-CS shows the advantages of using multi-objective optimization in clustering gene expression data.

Among the comparison algorithms, the performance of the PCFCMq algorithm, which is based on fuzzy clustering, is generally better than the hard clustering-based COP-Kmeans, PCKMeans, and MPCKMeans algorithms. According to (Gasch and Eisen, 2002), genes may be co-expressed with different genomes under different measurement conditions, and there is usually overlap between gene clusters. Therefore, compared with hard clustering algorithms, fuzzy clustering algorithms are more suitable for analyzing gene expression data. Furthermore, due to the proposed constraints selection and multi-source constraint fusion strategy, the MSC-CSMC algorithm achieves better clustering results than the PCFCMq algorithm. In terms of the robustness of the clustering results, the performances of semi-supervised clustering algorithms for comparison fluctuate with the increase of pairwise constraints, which is mainly due to the quality of randomly selected pairwise constraints. As stated by Lai et al. (2021), even non-noisy constraints that conform to the real cluster information may have a negative impact on the clustering results, which further illustrates the necessity of constraints selection in semi-supervised clustering algorithms. The proposed MSC-CSMC algorithm can select pairwise constraints suitable for clustering based on the co-evolution of the cluster prototype and constraints selection, which guarantees both accuracy and stability of the clustering results.

**FIGURE 5:**
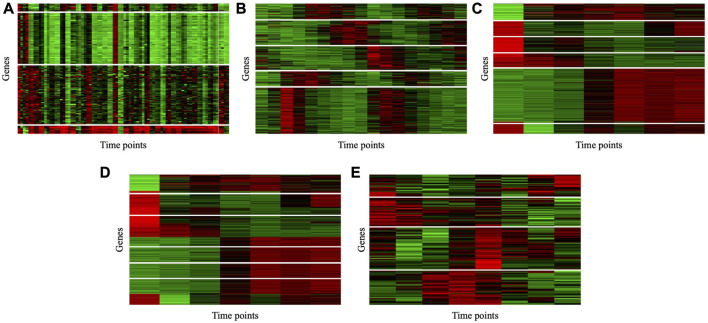
Eisen plots of the gene clusters obtianed by MSC-CSMC. **(A)** Yeast Galactose Metabolism with the number of constraints *s* =5 **(B)** Yeast Cell Cycle with the number of constraints *s* =10 **(C)** Yeast Sporulation with the number of constraints *s* =15 **(D)** Serum with the number of constraints *s* =20 **(E)** Arabidopsis with the number of constraints *s* =25.

**FIGURE 6:**
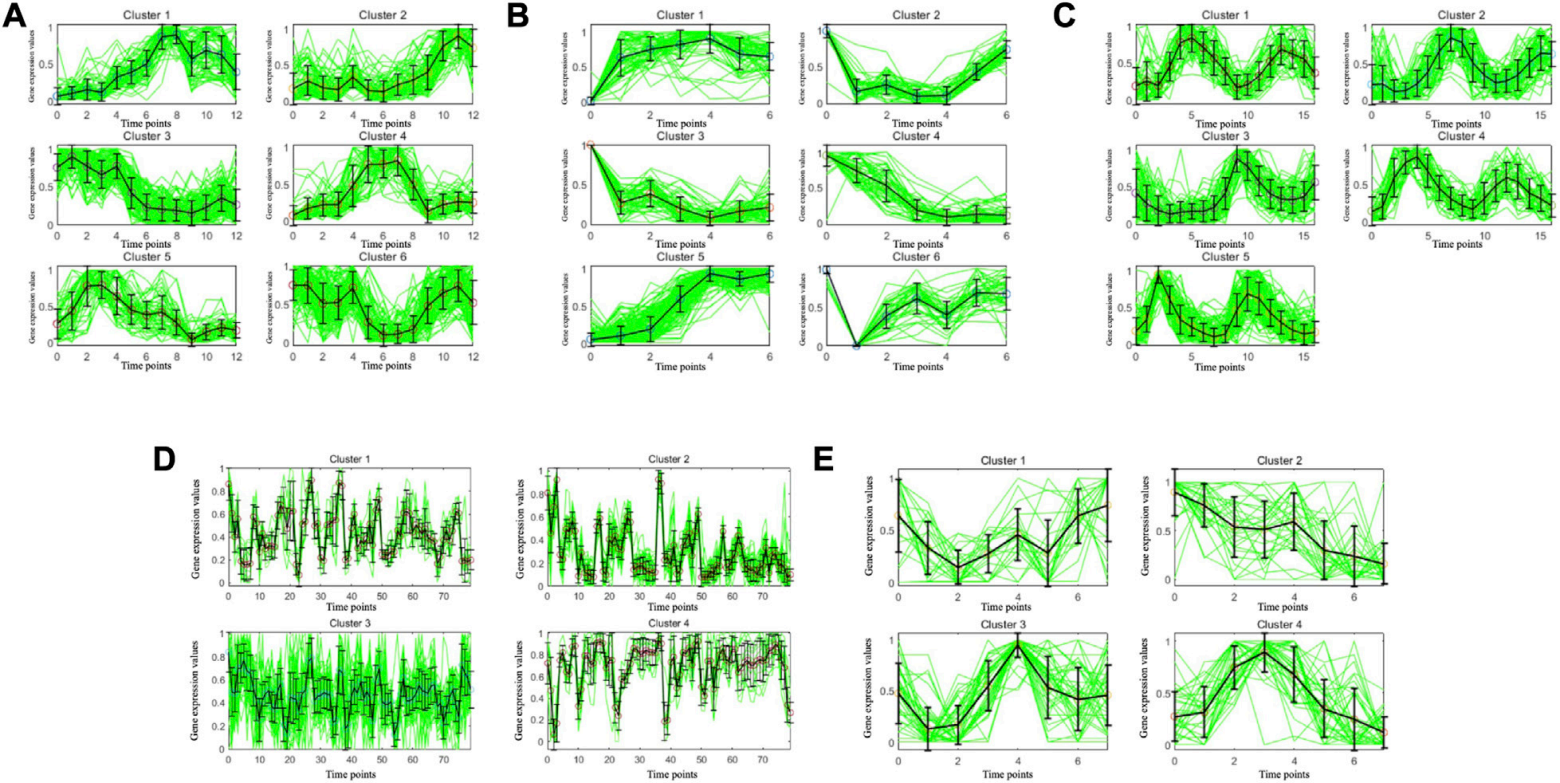
Cluster profile plots of the gene clusters obtianed by MSC-CSMC. **(A)** Yeast Galactose Metabolism with the number of constraints *s* =5 **(B)** Yeast Cell Cycle with the number of constraints *s* =10 **(C)** Yeast Sporulation with the number of constraints *s* =15 **(D)** Serum with the number of constraints *s* =20 **(E)** Arabidopsis with the number of constraints *s* =25.

To illustrate the consistency of the gene clusters obtained by the MSC-CSMC algorithm, the Eisen plots and cluster profile plots corresponding to the clustering results of five datasets are shown in Figure 5 and Figure 6. In the Eisen plots, each row corresponds to a gene, each column to a time point (sample), and each entry of the plot represents the expression level of a gene at a specific time point by coloring the corresponding cell. To illustrate more clearly the gene clusters obtained by MSC-CSMC, the genes partitioned into the same cluster are placed together. In the cluster profile plots, the X- and *Y*-axis represent the time points and gene expression values, respectively. The expression values of genes partitioned into the same cluster are plotted in the same subplot. In the subplots, each green line indicates the normalized expression values of a gene over all time points, and the black line represents the mean expression level of the genes in the corresponding cluster. It can be seen in the Eisen plots that the color patterns (expression levels) of genes in the same cluster are similar to each other, while genes in different clusters show different color patterns. According to Figure 6, the cluster profiles of different clusters are different from each other, and the cluster profiles within a cluster reveal consistency.

**FIGURE 7:**
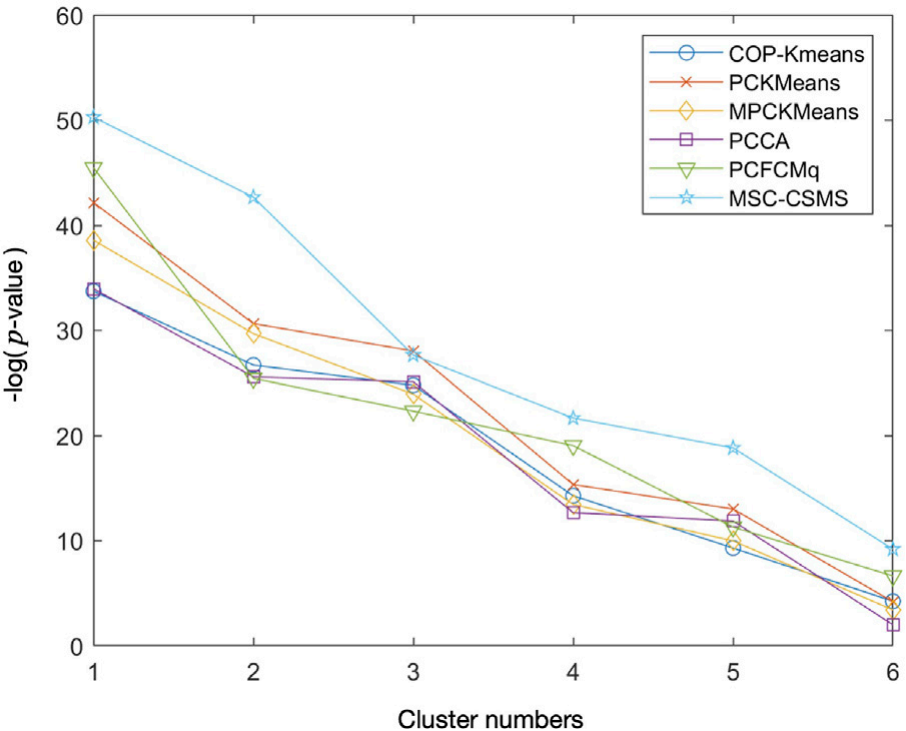
Average negative logarithmic *p*-values of the three most significant GO terms for each of the six clusters on Yeast Sporulation with the number of constraints *s* =15.

In order to inspect the biological significance of the gene clusters obtained by the MSC-CSMC algorithm, enrichment analysis is carried out using the GO annotation database, which results in the significant GO terms shared by genes in each cluster and their corresponding *p*-values. Taking the case where the number of pairwise constraints in the Yeast Sporulation dataset is 15 as an example, we focus on the three most significant GO terms (corresponding to the three lowest *p*-values) in each of the six clusters obtained by each algorithm. Figure 7 shows the plot of the average *p*-values. To illustrate the difference significantly, the *p*-values are negative log-transformed and the clusters are sorted in descending order according to the transformed values. Table 7 reports the three most significant GO terms and the corresponding *p*-values in each cluster obtained by MSC-CSMC.

**TABLE 7:** The three most significant GO terms and the corresponding *p*-values for each of the six clusters obtained by MSC-CSMC on Yeast Sporulation.

Gene cluster	GO term	*p*-value
1	meiotic cell cycle (GO:0051321)	1.42E-53
	meiotic cell cycle process (GO:1903046)	4.33E-51
	peptide biosynthetic process (GO:004304)	2.07E-48
2	sporulation (GO:0043934)	2.17E-45
	translation (GO:0006412)	4.08E-44
	sporulation resulting in formation of a cellular spore (GO:0030435)	1.02E-40
3	meiotic cell cycle (GO:0051321)	2.50E-30
	meiotic nuclear division (GO:0140013)	3.85E-28
	nuclear division (GO:0000280)	1.16E-26
4	cell cycle process (GO: 0022402)	7.37E-23
	cell cycle (GO: 0007049)	3.67E-22
	cell wall organization (GO: 0071555)	3.46E-22
5	cell development (GO: 0048468)	6.15E-20
	ascospore formation (GO: 0030437)	1.45E-19
	anatomical structure development (GO: 0048856)	3.53E-19
6	small molecule metabolic process (GO: 0044281)	2.51E-11
	amino-acid betaine metabolic process (GO: 0006577)	3.16E-09
	carnitine metabolic process (GO: 0009437)	3.15E-09

From Figure 7, it can be seen that the curve corresponding to MSC-CSMC is higher than those of the other algorithms, indicating that MSC-CSMC gains the result with the highest biological significance. Moreover, all the *p*-values of the significant GO terms listed in Table 7 are far less than 0.01, indicating that the MSC-CSMC algorithm can identify biologically relevant gene clusters.

## 4 Conclusion

Aiming at the problem that current semi-supervised clustering methods based on pairwise constraints are easily affected by noisy constraints and do not take the fusion of multi-source constraints into account, in this paper, we propose a multi-objective semi-supervised clustering algorithm based on constraints selection and multi-source constraints (MSC-CSMC). The proposed algorithm uses gene expression data and GO information to generate multi-source pairwise constraints and applies the multi-source constraints to the semi-supervised clustering process through improved constraint violation penalty weights. On this basis, a collaborative multi-objective optimization framework for constraints selection and cluster prototypes is constructed, and the negative impact of the noisy constraints is reduced by selecting pairwise constraints suitable for clustering. Experimental results on multiple gene expression datasets show that the MSC-CSMC algorithm effectively improves the performance of semi-supervised clustering. The validity of the proposed method proposed is not limited to the cluster analysis of gene expression data. Other semi-supervised clustering studies with multi-source information or constrained selection requirements can also be enlightened.

The effectiveness of the algorithm in this paper has been verified in small and medium-sized gene expression datasets. With the increase in the data size, the augment in the number of decision variables in the process of multi-objective evolution will lead to a decrease in algorithm efficiency and optimization performance. Therefore, the next step is to use decision variable analysis and other methods to design a multi-objective evolution strategy of the algorithm so as to further improve the applicability of the algorithm in practical clustering problems. In addition, we will also try to use various evaluation indices and design a multi-objective optimization framework with variable coding length (Rodríguez-Méndez et al., 2019) to optimize the number of clusters for gene expression data.

## Data Availability

The original contributions presented in the study are included in the article, further inquiries can be directed to the corresponding authors.
